# BioTM Buzz (Volume 3, Issue 3)

**DOI:** 10.1002/btm2.10116

**Published:** 2018-10-08

**Authors:** Aaron C. Anselmo

**Affiliations:** ^1^ Division of Pharmacoengineering and Molecular Pharmaceutics, Eshelman School of Pharmacy University of North Carolina at Chapel Hill Chapel Hill NC

## BioTM Buzz Volume 3, Issue 3

### Bugs secreting drugs

Recent interest in microbe based therapeutics have led to the development of novel approaches to deliver drugs for treatment of enteric infections. In this issue of Bioengineering & Translational Medicine, a team of researchers at General Probiotics in Minnesota describe an approach using genetically engineered *Escherichia coli* Nissle 1917 to both secrete and deliver a combination of antimicrobial peptides for the treatment of vancomycin resistant *Enterococcus*. The authors utilized a previously reported peptide expression approach that uses a Microcin V secretion system to enable secretion of the three antimicrobial peptides Enterocin A, Hiracin JM79, and Enterocin B. In this work, the Microcin V system was modified so that *Escherichia coli* Nissle 1917 could secrete higher amounts of antimicrobial peptides than previously reported. In a standard *in vitro* agar diffusion assay, it was shown that when *Escherichia coli* Nissle 1917 secreted these peptides individually, or in combination, significant inhibition of the growth of both *Enterococcus faecium* 8E9 and *Enterococcus faecalis* V583R was observed. It was further shown that the supernatant from the various *Escherichia coli* Nissle 1917 peptide‐secreting strains was enough to inhibit growth of both *Enterococcus* strains and that the combination of 3 peptides further improved inhibition as compared to the individual peptides. Significantly less *Enterococcus* was detectable in the feces of mice treated with bacteria‐secreting peptides as compared to untreated groups. One important aspect of this paper is the description and application of a novel microbe‐based system for the combination delivery of three synergistic peptides.

DOI: https://doi.org/10.1002/btm2.10107

### Electrospinning for scale‐up

Manufacturing biotechnologies at scale represents a significant challenge that can limit the translation of lab‐scale approaches, especially when they are not compatible with production‐scale equipment. Efforts to bridge this translational gap could improve and accelerate translation of cell‐based therapies, such as those used for tissue engineering applications. Highlighted in this issue of Bioengineering & Translational Medicine, engineers from Wayne State University describe an electrospray apparatus to encapsulate rat bone marrow mesenchymal stem cells in polyelectrolyte capsules at large scale. The authors characterize how polyelectrolyte choice, voltage, and needle size affect capsule diameter, morphology, and viability of the encapsulated stem cells. It was determined that increasing the voltage or decreasing needle diameter decrease resulting capsule size; however, it was also demonstrated in most cases that an increase in voltage initiated the formation of non‐spherical capsules. There are specific cases where either spherical or non‐spherical capsules are desirable, and as such toolbox development to understand how manufacturing conditions influence capsule shape are needed. The most important parameter governing viability at 30 days following encapsulation was determined to be polyelectrolyte choice and composition. It was hypothesized that capsules exhibiting the highest survival were those that facilitated optimal nutrient transport during their storage. In summary, this study highlights how key parameters can influence capsule properties that subsequently affect performance. Understanding ideal manufacturing conditions to improve biomaterial performance is a needed area of focus in translational research.

DOI: https://doi.org/10.1002/btm2.10111

## Recent literature

### Cell‐based modifications to improve delivery of synthetic systems

Recent work from the Liangfang Zhang lab in the Department of NanoEngineering at the University of California San Diego describes a nanoparticle‐based strategy for management of rheumatoid arthritis. In this work, neutrophil cell membranes were coated onto the surface of poly(lactic‐co‐glycolic acid) nanoparticles and in doing so the natural functions and properties of the neutrophils were transferred to the synthetic nanoparticle carrier. For example, through this membrane coating, the nanoparticles gained the abilities to bind proinflammatory cytokines and thereby inhibit chondrocyte activation and apoptosis. Furthermore, neutrophil membrane coated nanoparticles could penetrate into mouse femoral heads in nearly three‐fold higher amounts than control nanoparticles, which has implications in delivering these beneficial nanoparticles deep into difficult to reach tissues. When tested in vivo, the neutrophil coated nanoparticles exhibited significantly increased efficacy by reducing joint damage. This work highlights the potential in leveraging our natural delivery systems, our cells, to improve and perhaps enable the therapeutic benefits of synthetic technologies such as nanoparticles.

Zhang, Dehaini, Zhang, Zhou, Chen, Zhang, Fang, Gao, and Zhang, Nature Nanotechnology. 2018; DOI: https://doi.org/10.1038/s41565-018-0254-4

